# Methodology for comprehensive cell-level analysis of wound healing experiments using deep learning in MATLAB

**DOI:** 10.1186/s12860-021-00369-3

**Published:** 2021-06-02

**Authors:** Jan Oldenburg, Lisa Maletzki, Anne Strohbach, Paul Bellé, Stefan Siewert, Raila Busch, Stephan B. Felix, Klaus-Peter Schmitz, Michael Stiehm

**Affiliations:** 1 Institute for ImplantTechnology and Biomaterials e.V, Rostock, Germany; 2 Department of Internal Medicine, Cardiology, University Medicine Greifswald, Greifswald, Germany; 3 DZHK (German Centre for Cardiovascular Research), Partner Site Greifswald, Greifswald, Germany

**Keywords:** Endothelial cells, Unet, CNN, Neural network, Wound healing, Cell scale, Population scale, Cardiovascular

## Abstract

**Background:**

Endothelial healing after deployment of cardiovascular devices is particularly important in the context of clinical outcome. It is therefore of great interest to develop tools for a precise prediction of endothelial growth after injury in the process of implant deployment. For experimental investigation of re-endothelialization in vitro cell migration assays are routinely used. However, semi-automatic analyses of live cell images are often based on gray value distributions and are as such limited by image quality and user dependence. The rise of deep learning algorithms offers promising opportunities for application in medical image analysis. Here, we present an intelligent cell detection (iCD) approach for comprehensive assay analysis to obtain essential characteristics on cell and population scale.

**Results:**

In an in vitro wound healing assay, we compared conventional analysis methods with our iCD approach. Therefore we determined cell density and cell velocity on cell scale and the movement of the cell layer as well as the gap closure between two cell monolayers on population scale. Our data demonstrate that cell density analysis based on deep learning algorithms is superior to an adaptive threshold method regarding robustness against image distortion. In addition, results on cell scale obtained with iCD are in agreement with manually velocity detection, while conventional methods, such as Cell Image Velocimetry (CIV), underestimate cell velocity by a factor of 0.5. Further, we found that iCD analysis of the monolayer movement gave results just as well as manual freehand detection, while conventional methods again shows more frayed leading edge detection compared to manual detection. Analysis of monolayer edge protrusion by ICD also produced results, which are close to manual estimation with an relative error of 11.7%. In comparison, the conventional Canny method gave a relative error of 76.4%.

**Conclusion:**

The results of our experiments indicate that deep learning algorithms such as our iCD have the ability to outperform conventional methods in the field of wound healing analysis. The combined analysis on cell and population scale using iCD is very well suited for timesaving and high quality wound healing analysis enabling the research community to gain detailed understanding of endothelial movement.

## Background

In the past decade enormous progress has been made in the field of cardiovascular research and device development. In particular the performance of stents and transcatheter aortic valve prosthesis has been proven in multiple clinical trials (reviewed in [1, 2]).

Endothelial healing after deployment of cardiovascular devices is particularly important in the context of clinical outcome. Healthy endothelial cells (ECs) line the inner wall of every vessel and are known to suppress inflammation, re-stenosis and thrombosis. Numerous studies points out that endothelial recovery following injury due to device deployment is one of the most important limiting factors in coronary healing after stent implantation [3–5]. Furthermore, it was observed that endothelial proliferation and functions are highly dependent on the surface material [6, 7]. Therefore, the restoration of a functional EC monolayer on the surface of implanted devices represents an essential therapeutic goal to avoid severe clinical complications [8, 9].

For in vitro investigation of re-endothelialization, migration assays, generating a defined gap between two cell monolayers, are routinely applied [10]. In general, research objectives and thus analysis methods of cell assays can be categorized into two different levels: population scale and cell scale [11].

The subsequent analysis on a population scale such as gap closure is based on temporal observations obtained by live-cell imaging techniques and basically compares the duration of wound closure, in response to different chemical or mechanical stimuli [10]. Population scale analysis distinguishes only between the cell monolayer as an entity and the wound. This approach can be implemented easily and is therefore widely used.

Particularly ECs are exposed to mechanical stimuli generated by the blood flow and as a result the process of wound healing comprises different aspects of cell motion such as directed migration of the border cells, autonomous random migration of the inner cells and coordinated cell motion within the endothelium [12, 13]. To distinguish between the different types of cell motion, analysis of cell velocity on an individual cell scale is essential and must thus comprise the influence of local hemodynamic conditions on migration and directionality of EC movement.

To date, manual methods are applied to either count single cells or track their movement over sequential images [10]. This method is simple but it is also very time consuming and leads to user-dependent results. Therefore, an automated detection and segmentation of the cells is one key factor to analyze cellular kinematics and thus gaining an understanding of how cells behave and respond to changes in their local environment.

Numerous attempts have been made, which address this problem using automated methods. However, automated analysis of live cell images are often based on gray value threshold or gray gradient methods, such as the Canny method [14]. These analyses are limited by their sensitivity to variations in contrast, brightness and noise. Furthermore, assay contaminations or air bubbles may lead to erroneous cell detection. To overcome these limitations additional manual post-processing steps of image analysis are required [11]. Consequently, the number of cells to be detected must be kept small.

Several research groups pointed out that the rise of deep learning algorithms, such as Convolutional Neural Networks (CNNs), offers promising opportunities for application in medical image analysis [15, 16]. CNNs, such as the U-net architecture, are known as state-of-the-art machine learning subgroup of deep neural networks and have already proven their immense potential for image segmentation [17, 18]. In fact well-trained deep neural networks even exceeded human experts [18]. Numerous U-net versions have been presented to address segmentation problems in the field of medical image analysis [17].

Here we present an intelligent cell detection (iCD) network based on deep learning approach for a comprehensive assay analysis to obtain essential characteristics for cell scale and population scale. The detection and tracking of individual cells based on iCD are the fundament for further analysis of the wound healing assay and prediction of wound closure. To evaluate our iCD approach, we compared the results of important metrics based on conventional methods such as Canny method and Cell Image Velocimetry (CIV) as well as manual detection methods in an in vitro dynamic wound healing assay to the results obtained by iCD. Therefore we derived results of relevant metrics on cell scale such as cell density and cell velocity from cell detection and tracking. In addition, edge protrusion and wound closure, which are often used as metrics on the population scale, were also derived from individual cell detection by iCD. With our robust CNN-based iCD approach we are able to bridge the gap between automated cell-scale and automated population-scale analysis.

## Results

Some research groups are working intensively on the automated evaluation of wound healing experiments. Recently, deep learning methods have also been evaluated for this application. Often, the focus is on tracking cells, generating cell paths and deriving information about cell interactions. For example, Ulicna et al. developed an analysis tool (DeepTree) consisting of two neural networks, one for segmentation (U-net) and the other for deciding the status of the cell, like mitosis and apoptosis [19]. Other groups focus more on segmentation accuracy by developing and establishing new network structures [20–23]. They derive metrics from cell experiments that can be evaluated using positional information from individual cells. But, information on population scale, particularly in wound healing, is lacking. Recently, Javer et al. published a different deep learning approach for analyzing scratch assays. They do not concentrate on the exact segmentation of cells but on the positional probability of the cell center for investigation of collective cell motion of scratch assays (DeepScratch).

**Fig. 1:**
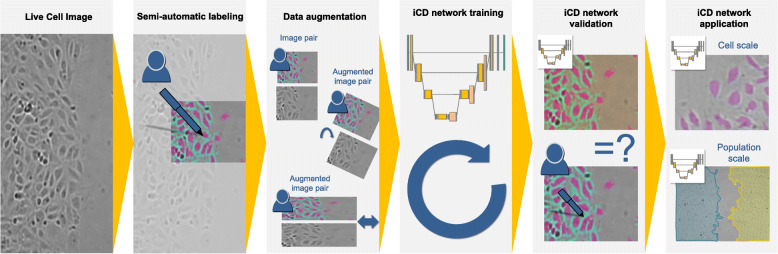
Workflow of deep learning MATLAB application for valid usage of iCD network. Live cell images from cell culture experiment were labelled manually supported by the implemented semi-automatic labeling module, which is based on the threshold method. Segmented image and the corresponding raw image form an image pair. By using rotating, scaling and skewing the image pair is augmented which creates a multiplied dataset for training. iCD Network is trained by using the raw image and the manually (semi-automatic) labelled image as input. Validation is performed by using only raw images only as input to the iCD Network and comparing the results with manually (semi-automatic) labelled image. After successful iCD training and validation, the network can be applied to novel live cell images to analyze cell motion at cell scale and population scale

We present a deep learning MATLAB application for investigation of live cell images on cell scale and population scale. For demonstration a wound healing experiment under different flow conditions (1.5 dyn vs. 10 dyn) was used. The workflow of the MATLAB application is shown in Fig. 1.

The MATLAB application consists not only of the U-net based iCD module but also of a training module. The training module starts with semi-automatic labeling of live cell images from wound healing assays. For this purpose, we created an algorithm with an adaptive thresholding method for segmenting cells and manually processing incorrect or inaccurate segmentations. This labeled data can be augmented to increase the training data and decrease the number of semi-manual cell labels needed. For creating the iCD network, we used a U-net architecture developed by Ronneberger et al. [24]. Using the trained network, we focus on segmenting each cell as accurately as possible. The segmented cell images were used for different application on cell scale (spatial distribution of cell density and cell velocity) and population scale (leading edge detection and wound closure analysis).

### Cell detection and segmentation

Cell detection is the initial step for further analysis presented in this paper. Live cell images of wound closure were obtained every 15 min over a period of up to 10 h. For validation of the iCD training we calculated the intersection over union (IoU), and the Boundary overlap ratio (F1-score) using 42 cell images with at least five cells at each frame. Overall 1467 individual cells were manual segmented. The IoU and F1 was computed for each of the Images using the MATLAB function ‘jaccard’ and ‘bfscore’. The mean IoU reaches a value of 0.8214 ± 0.038 and the F1 score 0.9178 ± 0.045. Manually, an average of 34.93 cells were detected on the validation images, while the iCD method detected 32.83 cells on the validation images (*p*-value: 0.644). Therefore, there is no statistically significant difference in the number of cells counted.

The adaptive threshold method is based on the local grey value distribution. Therefore, we manually defined a certain region of interest as well as a threshold sensitivity, which means that this method cannot be considered user independent. An area of interest of 161 × 121 pixels and sensitivity 0.5–0.7 were suitable parameters. To assess differences between the described methods, we applied the different cell detection methods (iCD, adaptive threshold method and freehand detection) to calculate the initial cell density in a dynamic wound healing assay using human coronary artery endothelial cells (HCAECs, see Cell density section).

### Cell density

**Fig. 2:**
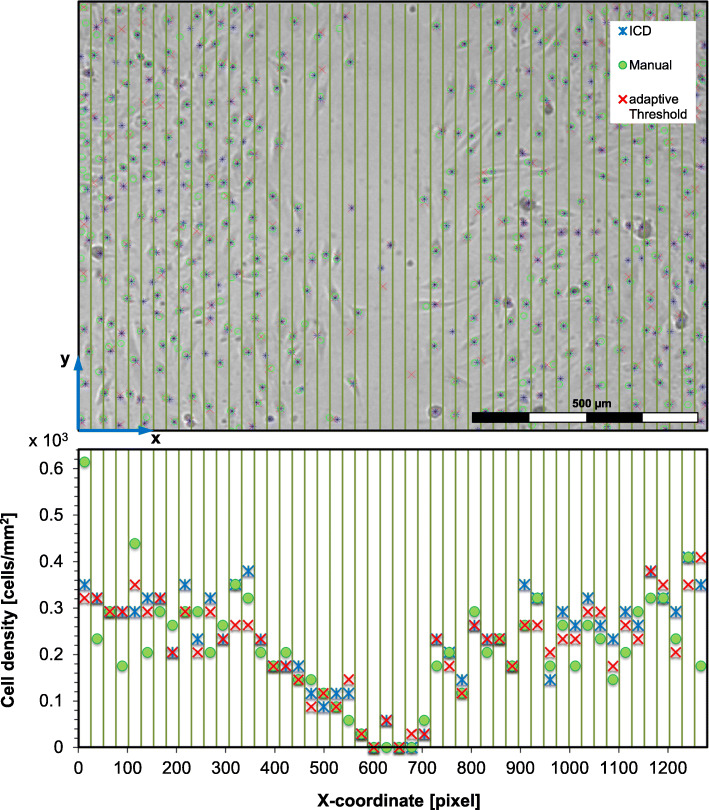
Detection of initial cell density by means of adaptive threshold method, intelligent cell detection (iCD), and manual detection. HCAECs were seeded in a 2-well insert (ibidi) and grown to confluency. Subsequently, the insert was removed to generate a defined gap between two monolayers of HCAECs. Over a period of 15 h gap closure was monitored by live cell imaging under dynamic conditions applying a shear stress of 1.5 Pa. **a** Shown here is a representative image of gap closure at t = 7.5 h after removal of the insert. **b** The cell density [cell counts / mm] was obtained by column wise averaging of the Y-coordinate of the live-cell image. The manually marked cell centers and the corresponding cell density distribution are in green, the iCD detection is in blue and the adaptive threshold detection is plotted in red

We compared different cell detection methods (iCD, adaptive threshold method and freehand detection) regarding their suitability for cell density assessment. For cell density analysis each image was divided into 50 columns, analogous to Jin et al. [10]. The number of cells was counted in each column and the total number of cells, per column was divided by the column area to obtain the cell density for each column. For illustration of the quality of the different approaches Fig. 2 exemplarily presents detected cells of a cell culture experiment.

For adaptive threshold we observed an overall relative error in the total cell count of 1.6% and for our iCD approach 8.5%. The relative error refers to manual data.

**Fig. 3:**
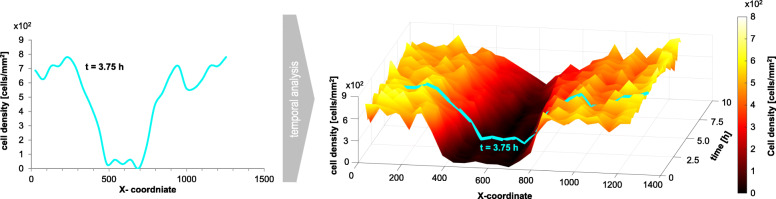
The cell density distribution for the time step t = 3.75 h (left) and 3D illustration of the temporal process (t = 0 h to 10 h; 40 live cell images) of wound closure according to cell density distribution. Cell density was obtained by using iCD method

To evaluate the temporal wound healing process, the column-averaged cell density, obtained by the adaptive threshold method and by the iCD approach, was plotted for each time point. Exemplarily Fig. 3 depicts the results obtained by iCD (t = 0 h up to 10 h, ∆t = 15 min).

**Fig. 4:**
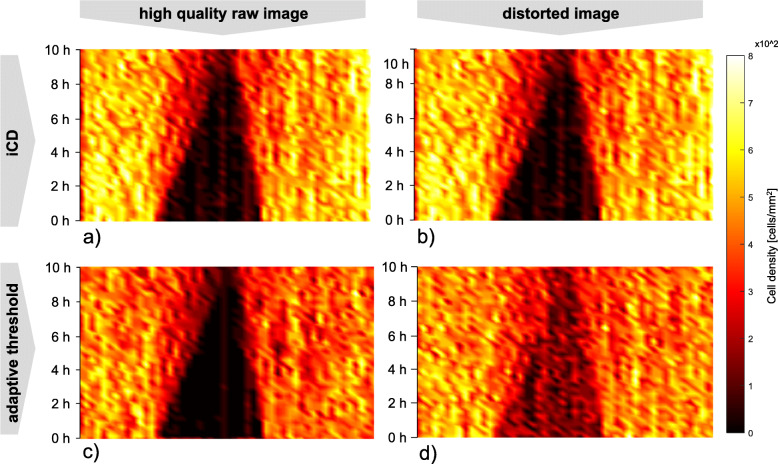
Temporal and spatial cell density distribution. **a** ICD **b** ICD with noise **c** adaptive threshold **d** adaptive threshold with noise

The live cell images were of high quality (Fig. 2) regarding noisiness. Next, the robustness of these two methods against image distortion was tested by manipulating high-quality raw images applying the MATLAB Gaussian noise filter. The analysis of cell density was repeated by means of adaptive threshold method and iCD using the distorted images (Fig. 4).

In general, both methods showed similar cell density values and distribution when using high-quality raw images (Fig. 4a and c). The asymmetry of the gap closing is caused by the use of live cell images under flow conditions. By applying the MATLAB Gaussian noise filter, the cell density of the scratch obtained from the adaptive threshold method differs from the former results using the high-quality raw images (compare Fig. 4c and d). Especially in the region of the gap, where no cells are present, noisy images lead to erroneous cell detection. However, the results of the iCD method were barely influenced by image manipulation as shown in Fig. 4b compared to adaptive threshold (compare Fig. 4a).

### Cell velocity

**Fig. 5:**
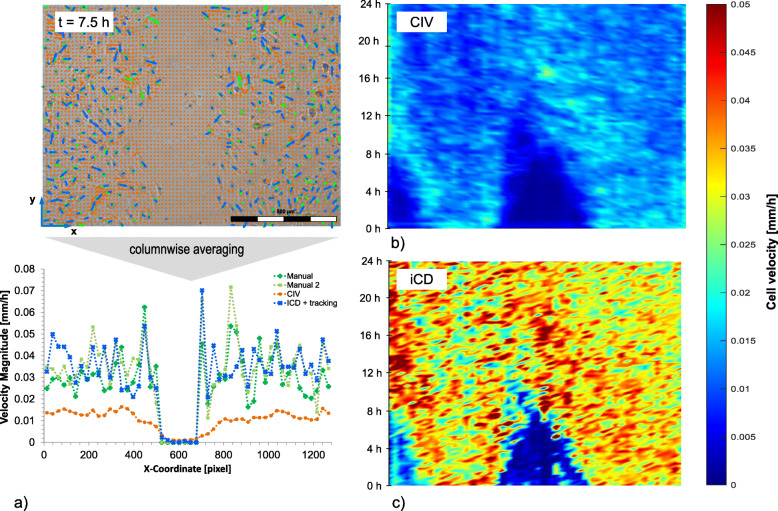
Spatial and temporal cell velocity: **a** Velocity field of EC at time step 7.5 h and **c** Column-wise averaged velocity magnitude for iCD method (blue), CIV method (green) and Manual (orange and red) **b** Temporal and spatial velocity magnitude distribution in mm/h for CIV method and d) ICD method ranging from 0 h to 24 h

We compared different approaches to validate the detection of cell motion: adaptive cell image velocimetry (CIV), iCD-based cell tracking and manual tracking of individual cells (Fig. 5a). The latter was used as reference. Since the gap between the cell monolayers is positioned perpendicular to the x-direction, averaging characteristics in the y-direction (column-wise), such as the cell velocity, are assumed to be valid. Based on the velocity field of ECs column-wise averaging of the velocity magnitudes was performed, resulting in a velocity function, which only depends on the x-direction. As reference, the cell velocity was investigated three times by manual cell tracking for one time step (at time point t = 7.5 h, ∆t = 15 min); Fig. 5a). This time step was selected because the wound healing was well established and therefore all cells were set in motion, even at a distance from the gap.

We obtained an average velocity magnitude of 0.031 mm/h with an absolute and relative error between different manual analyses of 7 mm/h and 22%, respectively. The relative error refers to manual data. The average velocity detected by CIV was 0.012 mm/h with an averaged absolute and relative error of 0.020 mm/h and 62.7% compared to manual tracking. The average velocity detected by iCD was 0.036 mm/h with an averaged absolute and relative error of 0.0047 mm/h and 14.5%, compared to manual tracking.

To investigate the cell velocity during the healing process we applied the adaptive CIV and iCD method on every live cell image (t = 0 to t = 24 h). The results are plotted in Fig. 5b and c. A difference between the cell velocities resulting from iCD and CIV was also observed over time. For example, a maximum cell velocity of > 0.05 mm/h was determined using iCD, whereas the highest cell velocity values for CIV were 0.03 mm/h.

### Leading edge detection and wound closure analysis

**Fig. 6:**
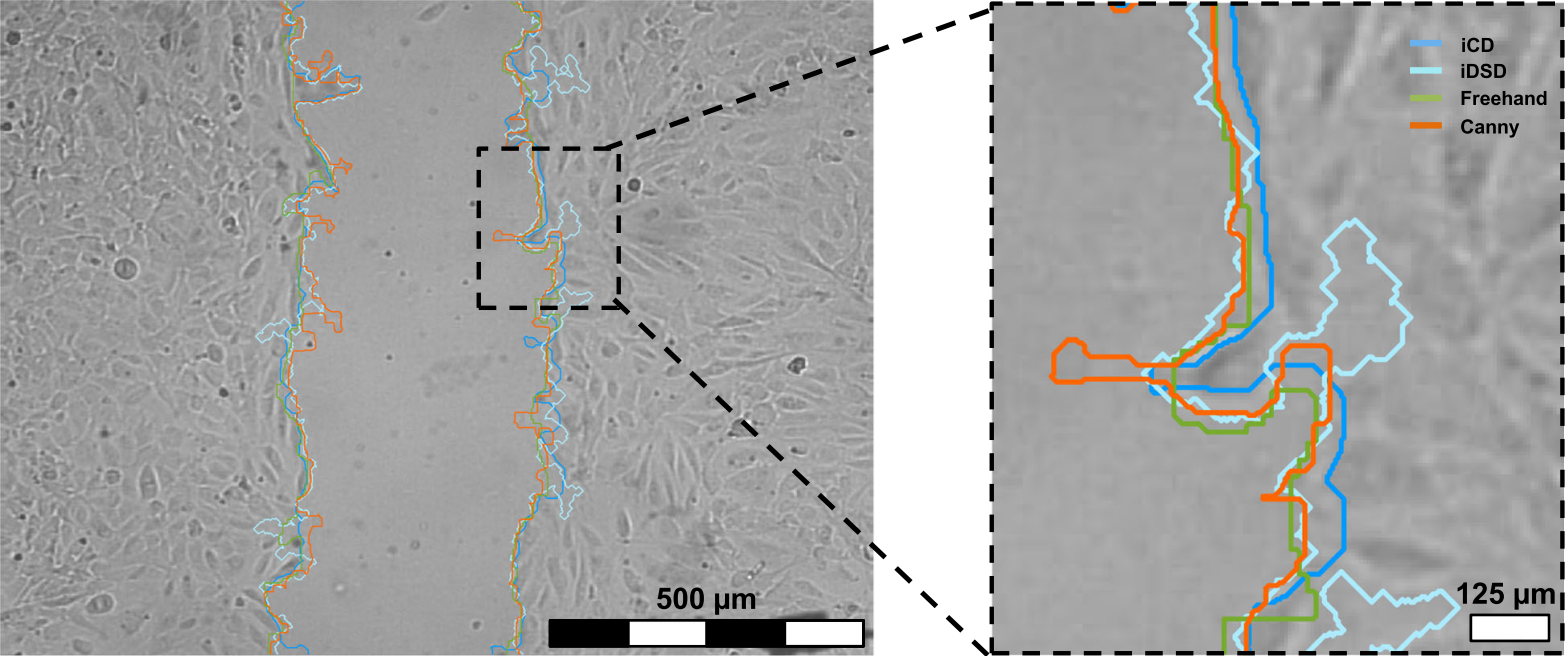
Leading edge 7.5 h after starting of wound healing assay. The predicted leading edge using conservative Canny method is plotted in orange, as well as from iCD in dark blue, the iDSD in blue and freehand in green

Leading edges were detected by the conventional Canny method (orange), by a second deep learning approach: called intelligent direct scratch detection (iDSD) (light blue), iCD (dark blue), and manual (green), see Fig. 6 (time point = 3.75 h). The leading edge obtained by Canny method and iDSD show a tendency to become more frayed, while the contour obtained from the iCD method is in better alignment to the freehand line.

**Fig. 7:**
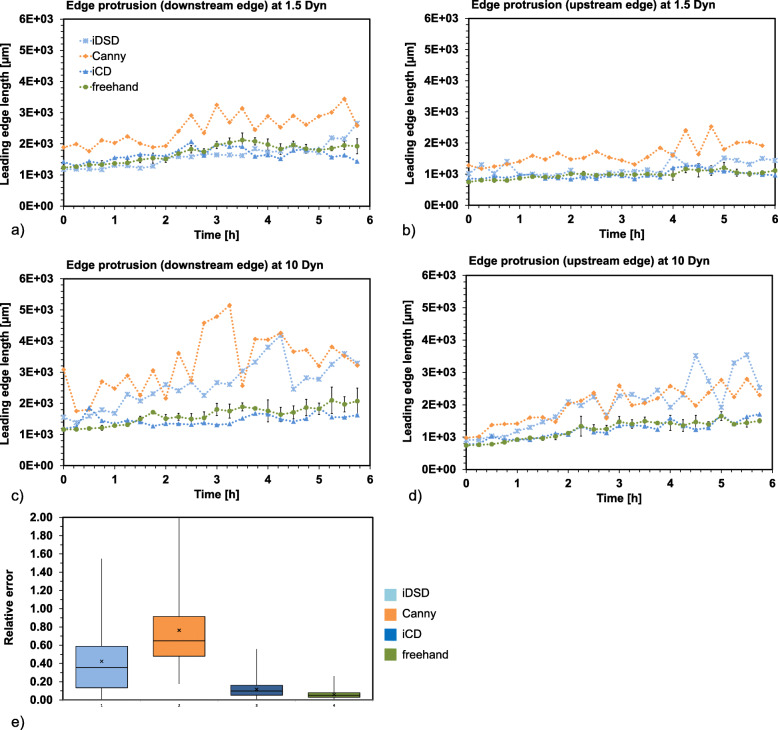
Edge protrusion of the upstream and downstream edge under different flow conditions 1.5 Pa and 10 Pa. **a** Protrusion of the downstream and **b** upstream leading edge at 1.5 Pa and **c** downstream and **d** upstream leading edge at 10 Pa using conservative Canny method (orange), as well as iCD (dark blue), iDSD (blue) and freehand (green) **e** Relative errors plotted as boxplot diagram compiled from a-d. The median is plotted as a vertical line in the box The upper box bound corresponds to the median of the upper half of the data and so also for the lower part. The mean is shown as a cross. The length of the whisker corresponds to 1.5 times the distance between the lower and upper box boundary; outside this range, the data are marked as outliers (circle)

A quantitative evaluation of the leading edge detection can be achieved by the highly sensitive edge length or so-called edge protrusion. Again, we compared the Canny method, our two different CNN approaches (iDSD and iCD) against the freehand edge as reference. The edge length was measured every 15 min over 6 h. In Fig. 7(a-d) we plotted the absolute edge length of both upstream and downstream edges. Manual data were obtained three times for each time frame. The average values at each time point is displayed in the diagram, with its corresponding standard deviation and is used as reference for the relative error computation. For statistical analysis the relative errors of leading edge protrusion are compiled in a boxplot shown below (Fig. 7e).

The relative error in the determining of the leading edge protrusion is lowest for freehand values, but even here a relative error of 5.9% is found on average. Referencing the freehand line, the iCD method has the lowest relative error regarding edge protrusion (11.7%) and has the smallest variation around the mean value. An average relative error for left and right edge protrusion of 76.4% were found using the Canny method and 42.4% using the iDSD approach. All methods detected an increasing edge length, which indicate the migration of pioneer cells into the wound. In general, the Canny method overestimated the edge length compared with freehand detection.

**Fig. 8:**
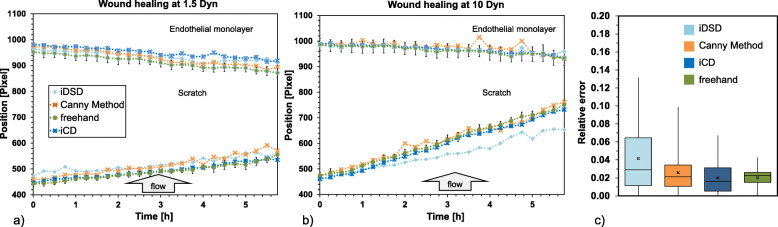
Spatial averaged edge position calculated at different time steps, depicts the wound closing rate at **a** 1.5 Pa and **b** 10 Pa. Using conservative Canny method (orange), as well as iCD (dark blue), iDSD (blue) and freehand (green) **c** Relative errors plotted as boxplot diagram compiled from **a** and **b**. The median is plotted as a vertical line in the box The upper box bound corresponds to the median of the upper half of the data and so also for the lower part. The mean is shown as a cross. The length of the whisker corresponds to 1.5 times the distance between the lower and upper box boundary; outside this range, the data are marked as outliers (circle)

The wound healing process can also be quantified by the position of the leading edge as a function of time. Therefore, based on the former edge detection the spatially averaged edge position was calculated at different time steps (Fig. 8a, b) using Canny, iDSD, and iCD method in comparison to the freehand analysis (3 independent datasets were used). For statistical analysis the relative errors of spatially averaged edge positions are compiled in a boxplot shown below (Fig. 8c).

All methods detect a comparable wound healing behavior. It can be seen, that under low flow (1.5 Pa) the upstream edge is slightly faster than the downstream edge. The difference is more pronounced with increasing flow (10 Pa). Even if the Canny method is distorted by imaging artefacts, the edge position is in a very good agreement with the manual detection due to spatial averaging. The iDSD localized upstream edge behind the edge positions, which were obtained by other methods. The reason for this is shown in an example image, where the direct detection of the edge works very well in the beginning and can lead to false detection in the further time through changes of the image quality (Fig. 8). When determining the spatially averaged edge position, the iCD method shows the same mean relative error (2%) as freehand, but a slightly higher variation. Both the Canny method (2.6%) and the iDSD (4.2%) approach showed higher relative errors in the determination of the spatially averaged edge position.

## Discussion

Here we present a novel approach based on deep convolutional neural networks – called intelligent cell detection (iCD) – to enable a reproducible, user-independent method for the accurate evaluation of in vitro wound healing assays both in population as well as cell scale. To evaluate the suitability of our iCD approach, we compared results based on currently used adaptive and manual detection methods in an in vitro dynamic wound healing assay to the results obtained by iCD. Overall, iCD provides user-independent results regarding cell density, cell velocity, leading edge detection, edge protrusion, and wound closure with a substantially lower susceptibility to errors than comparable methods.

### Cell detection and segmentation

**Fig. 9:**
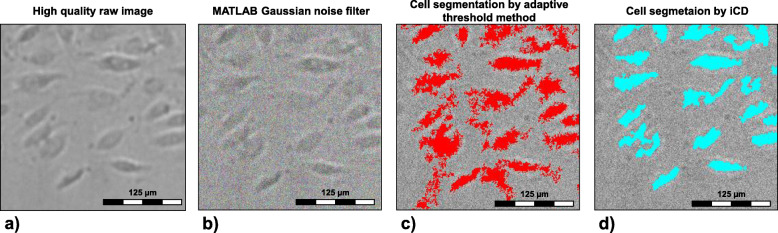
Segmentation example under image distortion, **a** original Image **b** image with applied Gaussian noise **c** segmentation using adaptive threshold **d** segmentation using iCD approach

The utilized U-net architecture has previously been proven to be highly suitable for CNN-based segmentation [24], which is further confirmed by the present study. It is of particular benefit, that only very few annotated images are needed to train the network on the one hand owing to the structure of the network containing feature concatenation (bridging) of the down and upward convolutions and on the other hand due to excessive image augmentation. We successfully trained the neural network and scored IoU values that are comparable to those in literature. The IoU reaches a value of The mean IoU reaches a value of 0.8214 ± 0.038 and is in a range of the IoU values presented by Ronneberger et al. (IoU = 0.9203 for segmentation of Glioblastoma-astrocytoma U373 cells and 0.7756 for segmentation of HeLa cells) [24]. The results showed that our iCD method is performing as good as conventional methods such as adaptive thresholds methods when using high quality images. To demonstrate the superior segmentation by iCD, we tested our method using distorted cell images. Exemplarily, Fig. 9a show a live cell raw image, the artificially distorted image (Fig. 9b) and the resulted segmentation utilized the adaptive threshold method (Fig. 9c) and our iCD method (Fig. 9d).

It should be noted that the outlined method can also be applied to wound healing experiments with any other cell line. Other microscopic cell experiments, involving population and cell scale correlations, can also be analyzed.

There are two options using the pre-trained MATLAB application that will be uploaded on MATLAB file exchange. Option 1: If the pre-trained network achieves sufficiently good segmentation results, the network does not need to be retrained and the user can apply the pre-trained software implementation. Option 2: If the segmentation results are insufficient, the network can be re-trained and re-validated. The user is supported by the implemented semi-automatic training module, which is based on the threshold method.

### Cell density

Local cell density is a crucial quantitative metric for the evaluation of cell migration and proliferation [6] and is therefore used to describe the progress of wound healing. In this context, wound closure is highly sensitive to the initial cell density. It has been shown, that the detection of the initial cell density could enhance the reproducibility of wound healing assays [10]. We were able to prove that CNN-based cell density analysis is superior to an adaptive threshold method, particularly when the image is distorted. The obtained temporal cell density function could be useful for parameter identification for future analysis by the Fisher Kolmogorov approach [25].

### Cell tracking and cell velocity

Detailed information about collective cell migration is necessary to enhance the biological understanding of wound healing, cancer invasion as well as embryonic morphogenesis and tissue remodeling [26]. The former analyzed cell density only provides information of the cell distribution along wound healing process but does not describe the cell motion. Franco et al. pointed out that the velocity depends on the cells position regarding the wound, on the shear forces applied and on the topography of the substrate [27]. As consequence, not only the velocity value but also the directionality of the cell motion is a relevant factor for detailed cell analysis. Thus, we compared the results obtained by the commonly applied CIV tracking with the results obtained by iCD-based as well as manual tracking. Due to the principle of the CIV method, it is possible to measure collective cell movements by using adaptive correlation as an established post-processing routine for PIV, which was former developed for fluid flow measurements. Recently, this routine has also been used for non-hydro applications such as cell velocity measurement [28–32]. We tested the commercially available adaptive PIV algorithm from Dantec Dynamics. However, open source software is also available, [33] and has produced comparable results in additional tests. Our data show that the column-wise averaged cell velocity determined by CIV differs from the results obtained by iCD and manual tracking by a factor of 0.5. These findings are in accordance with assumptions in current literature, which states that the CIV method does not detect the physical velocity of the cell monolayer, since deformation and proliferation of the cells are also mistakenly considered as cell motion [32]. But they assumed a valid impression of the monolayer movement, since the errors occur in all directions. Furthermore, directionality as well as velocity gradients of the monolayer can be derived directly from the CIV dataset. It has to be noted that trajectory of individual cells cannot be investigated because the grey value shift of the substrate in a certain interrogation area is interpreted as velocity and not the motion of individual cells [29].

For iCD-based cell tracking we used the nearest-neighbor-algorithm. This cell tracking algorithms are often extended by a prior estimation of the future position of the cell using kinematic models. However, in our case we have not found any benefits of this extended method, because the movement of the cells is often not continuous over time and so the estimation of the future position is limited. If individual cells cannot be detected consequently for every live cell image, it is possible that the tracking algorithm links false cells. One can partly counteract this failure by entering a maximum length of cell motion (R) during one time step, which is recommended to the user. When selecting the maximum length of cell motion, care should be taken to ensure that the length is not too small to prevent a systemic threshold of larger cell velocity. Based upon the evaluation of the manual tracking of our cell experiments we set R between 25 and 30 Pixel, corresponding to a velocity between about 0.14 mm/h to 0.17 mm/h. The resulted velocity range is slightly above the maximal cell velocity (< 0.1 mm/h). This ensures that the cell moves within the previously defined radius. Our results showed good qualitative and quantitative agreement with our manual tracking of cells. Despite the fact that we used the particle tracking algorithm provided by Dantec Dynamics, we would like to mention that any other tracking algorithm can be applied as well, such as the MATLAB implemented algorithm by Tinevez et al. [28], which gave us very similar results.

### Wound closure analysis

We compared the common Canny method for population-scale analysis of EC monolayer with respect to edge protrusion and gap closure to our iCD approach and manual tracking. A valid detection of the leading edge is an important population-scale metric [11]. In particular, the edge protrusion is useful metric to evaluate the influence of pioneer cells on wound healing under various flow conditions [27]. An attempt to directly detect the gap and differentiate it from the cell monolayer, using U-net based direct scratch detection (iDSD), shows no advantage over the conventional method. The iDSD as well as the Canny method are not able to distinguish between artefacts due to contamination or image distortions and the EC monolayer. So pollution and air bubbles can lead to false detection. Especially when the gray scale distribution and the gradient of the artifacts are comparable to the EC, both methods are prone to errors. This differs from the iCD method, in which the individual cells are segmented and not only the monolayer as an entity. Furthermore, the iCD post-processing routine automatically removes small elements, such as artifacts and contaminations, with improbable cell size. In conclusion the detection of the leading edge based on individual cell detection by our iCD approach gave results which are in better agreement with the freehand detection than the commonly applied Canny method and iDSD approach (Figs. 7, 8, 9). The iCD method allows a comprehensive analysis of the motion of the cell front or leading edge and provides essential information on frequently asked questions: (i) How does the geometry of wound affect the velocity of the leading edge? [11, 34], (ii) How does mechanical stress affect the velocity of the leading edge? [27] or (iii) How does cell density affect the velocity of the leading edge? [10].

## Conclusions

Setting up in vitro live cell imaging experiments itself can be challenging as cells die, air bubbles or pollutants are trapped or objects in the image are difficult to distinguish from the background. This means that the researcher may not always achieve a high image quality for further analysis. But currently applied methods for analysis of wound healing assays are prone to errors especially for heterogeneous background brightness, variable lighting conditions and similar gray scale distribution of object and background. Deep learning algorithms, such as CNN, promise to overcome the limitations of currently applied methods. In a CCN-based iCD analysis of a wound healing assay, we demonstrate that this approach is superior to a commonly applied adaptive threshold method in terms of robustness against image distortion. Furthermore, we post-processed the results of individual cell tracking for population-scale analysis such as edge protrusion. The obtained results are in very good agreement with freehand edge detection. The Canny method, a commonly used conventional threshold method, leads to higher protrusion values due to false cell edge detection. The attempt of direct scratch detection using an U-net architecture also showed too high protrusion values. Up to now, researchers manually tracked individual cells in tedious, time consuming studies to obtain information about the behavior of cells on the cell-scale, or they developed new strategies to obtain information about the population-scale in an automated way. With our robust CNN- based iCD approach we are able to close the gap between automated cell-scale and automated population-scale analyses.

Our approach outperformed conventional methods and is therefore feasible for comprehensive wound healing analyses and provides spatial and temporal resolved information of the endothelialization process. Such an approach is currently needed for the development of next generation cardiovascular implants with improved endothelial recovery.

## Methods

### Wound healing experiments

Human coronary artery endothelial cells (HCAEC) were purchased from Cell Systems, Germany. To evaluate initial cell growth, HCEAC were used at passage 3 to 5 and cultured in endothelial cell growth medium (Cell Systems, Germany) containing 10% fetal calf serum (FCS). Cells were seeded with a density of 3 × 10^5^ cells/mL in Culture-Inserts 2 Well (Ibidi, Germany) on a 25 × 75 mm Thermanox™ coverslip (fisher scientific, Germany). According to the manufacturer’s instructions, 70 μL of the cell suspension was added to each well. After 24 h the insert was removed, thereby generating an accurate longitudinal gap between both monolayers of approx. 500 μm. Subsequently, the coverslip was attached to a 0.8 mm sticky-slide I Luer perfusion channel. Using the ibidi pump system (Ibidi GmbH, Germany), endothelial cells were exposed to a constant laminar wall shear stress of 0.15 Pa or 1 Pa in an incubator (5% CO_2_, 95% H_2_O). Additionally, control samples were kept under static conditions. In the incubator, cell movement was monitored over a period of 15 h using the JuLiTM Life Cell Analyser (NanoEnTek, Korea). Every 15 min a live-cell image was captured. Since this work is intended to serve as a basis for future studies on cell growth under flow conditions, all results presented here are based on wound healing assays under flow.

### Cell detection and segmentation

**Fig. 10:**
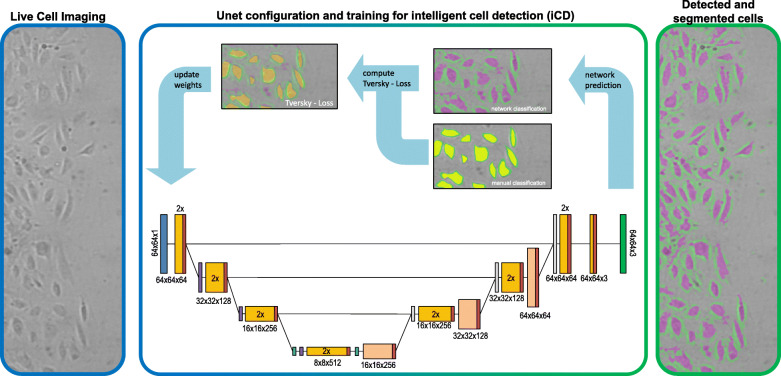
Schematic representation of the network training of the used U-net architecture to segment live cell images of endothelial cells in wound healing experiments. The network is composed of 3 down-sampling convolutional steps and 3 up-sampling stages of de-convolutional layers, the dimensions and number of feature layers of the steps is noted below the blocks (x-dimension, y-dimension, feature layers). The coloring of the blocks stands for: Input Image (blue), convolutional layer (orange), de-convolutional layer (apricot), max-pooling (purple), drop out (cyan), rectifier layer – ReLU (red), classification output (green). The loss during training is computed due to Tversky-loss function. The final segmentation results are pixels that are classified in cell, border or background

In order to segment the endothelial cells we used the U-net semantic segmentation network, developed by Ronneberger et al. 2015 [24]. Here, we utilized a variation of the network implemented in MATLAB consisting of 3 encoder and decoder stages. Each contraction stage consists of two convolutions (3 × 3) with a linear activation function (ReLU) followed by 2 × 2 maximal pooling layer. The stages of the upsampling side of the network consist of transposed convolutions and the concatenated feature maps from the corresponding downsampling path as well as following ReLU layers [24, 35], see Fig. 10.

The Network structure was generated by using MATLAB’s pre-implemented function unetLayers. For more detailed descriptions of the network, refer to Ronneberger et al. 2015 and to the MATLAB documentary. To classify each pixel we used a softmax layer. The network loss during training was calculated by Tversky-Loss function, which balances false positive and false negative detections (weighting factors: alpha, beta). Therefore, the MATLAB implementation of Salehi et al. was used, whose work is recommended to gain a detailed understanding [36].

Using U-net, a wide range of image sizes can be segmented. The only limitation of the segmentation process is that the edge lengths of feature maps must be even before applying the max-pooling. To use any image size the image needs to be mirrored at the edges and cropped to the required size. Mirroring at the edges also has the advantage that cells at the image border can be segmented without artifacts [35].

To separately segment cells that are close to each other cells and cell border were labelled separately as described by Ronneberger et al. 2015 [24].

For Training we semi-manually segmented cell images using an adaptive thresholding algorithm and manually fine-tuned the predictions. Therefore, a customized graphical user interface was programmed, which easily allows the user to generate additional training images in order to improve the segmentation result of the network on specific cell images. In this way 280 arbitrary sized training images were segmented. To train the network sufficiently with few training images, it is absolutely necessary, to perform data augmentation. In each epoch we generated up to 100 augmented images from one original training image by applying random skewing, rotation, translation and brightness variation. The augmentation of the images was performed on the fly, using the *imageDataAugmenter* function in MATLAB.

In order to solve the minimization problem we used an adaptive momentum solver with a learning rate of 0.001. As one of the most important hyperparameter for the training; the learning rate was reduced by multiplying 0.9 in every epoch. Before training, the image data were normalized between 0 and 1. Training worked out well with a small mini-batch size of 30 images. To avoid that the network is only learning structures from the last training images, the data was shuffled before each epoch. As weighting factor for the Tversky-Loss we achieved the best results with alpha = 0.3 and beta = 0.7. The network was trained on an Nvidia GTX 1500i with 4GB GDDR5 RAM with Cuda 10.2.

The output of the network is an image, which is segmented into background, cell and cell-border. We applied a median filter on the segmentation results to remove noisy predictions. For further processing the results were binarized with the background. Therefore, cell-borders were set to 0 and cells were set to 1. Accordingly, all connected pixel regions form an individual cell. To validate the segmentation results, Jaccard matrix was used and referred to as Intersection over Union (IoU) and the F1 score. This metric also penalizes false positives.

For conventional cell detection we applied an adaptive threshold method in MATLAB by using the Image Processing Toolbox (MATLAB R2019b). This threshold was adjusted based on the locally calculated mean grey value field. In our study, a size of 161 × 121 pixel was chosen as neighborhood for the calculation of the mean intensity. A sensitivity factor (ratio of background and foreground pixels) was set manually, which allows for an adaptation to the particular cell series. Using morphological operations, holes in detected cells were closed (*imfill*) and very small segmentations below the manual observed minimal cell size were removed (*bwareaopen*).

### Cell tracking and cell velocity

#### Cell tracking based on iCD

**Fig. 11:**
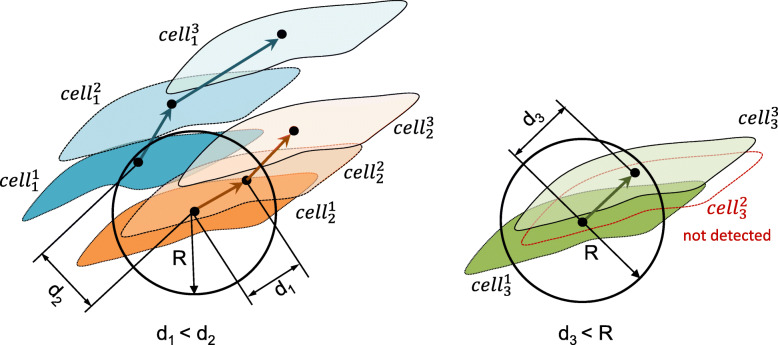
Scheme of cell tracking principle, of cell (i) at frame (t): ${\mathbf{c}}_{\mathbf{i}}^{\mathbf{t}}$: the distance between correct cell linking will optimally be found using nearest neighbor function (d1 > d2). For missing cell detections (see ${\mathbf{c}}_{\mathbf{3}}^{\mathbf{2}}$) an maximum length of cell motion (R) can be applied to validate the computed distance of linked cells (d3 < R)

By means of the iCD approach individual endothelial cells were detected on every live cell image. As a result we were able to define the position of an individual cell by the centroid of the cell area and each individual cell was assigned by a unique ID. The challenge of cell tracking lies in the recognition of individual cells in sequential images. For our cases we applied the so-called nearest-neighboring-algorithm, see Fig. 11. According to this method the nearest cell centroid on the following frame is assigned to the cell center of the previous frame. The quality of the algorithm was increased by defining a maximum length of cell motion (R), which is an additional criterion for cell recognition.

#### Cell image Velocimetry (CIV)

The Cell Image Velocimetry (CIV) approach originates from Particle Image Velocimetry (PIV) method which widely used in fluid mechanics [32]. PIV is a full-field post-processing method for the determination of velocity fields in fluid flows. For this purpose, correlations between small subunits, so-called interrogation areas (IA), of two sequential images were determined. From the displacement (grey value shift) and the time step between the sequential images, a velocity vector was obtained for each IA [29, 37]. Tracer particles which are usually required for flow visualization when using PIV are not necessary for CIV. Here, the movement of cell compartments and structures already results in an evaluable signal. We applied an adaptive correlation algorithm from Dantec Dynamics (Dantec Dynamics A/S, Denmark). This algorithm is included in the PIV-analysis software Dynamic Studio. The minimum and maximum size of the IA was defined as 32 × 32 pixels and 64 × 64 pixels, respectively. The permitted overlay of the IA was set to 50%. For this study, we used raw cell-images without any image preparation.

### Leading edge detection for wound closure analysis

#### Leading edge detection based on iCD

The iCD method provided individual segmented cells. In order obtain a closed cell front on the edge of the monolayer one needs to upscale the results from cell -scale to population -scale. Therefore each pixel of each cell boundary was radially dilated by factor 15, so that all cell boundaries were slightly overlapping. Afterwards, by using the *bwareaopen* function of MATLAB the two largest areas, the gap and the cell monolayer, were kept. Then both areas were eroded by a factor of 15. By means of the *bwboundaries* function we were able to detect the edge of the gap and calculated the edge protrusion as well as edge velocity in an additional post- processing step.

#### U-net for direct scratch detection

**Fig. 12:**
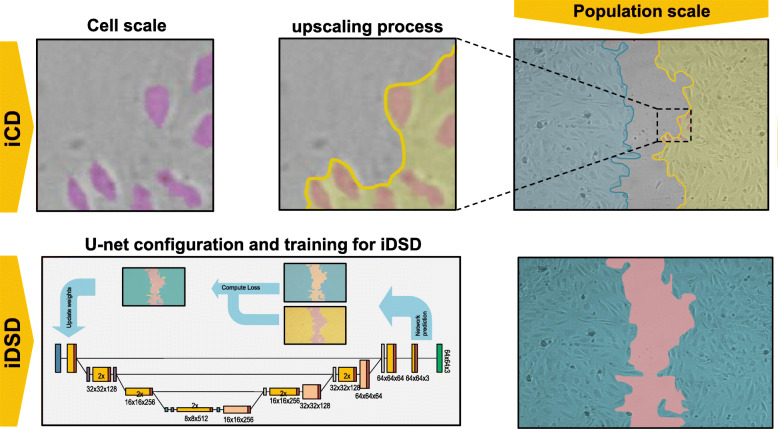
The figure shows the two approaches for the analysis of the population scale of endothelial cell scratch assays, on the one hand using the segmentation results (ICD) followed by morphological operations to determine the population scale and on the other hand the intelligent direct scratch detection approach (iDSD) which also uses the U-net architecture

As a second AI approach, our intelligent direct scratch detection (iDSD) approach aims to train a U-net on the population -scale level by using the manually segmented gaps. The network architecture corresponded to the one described above for iCD. A total of 170 training images were applied to train the network and again these dataset was augmented by the MATLAB data augmentation function as described before. In total 100 augmented images per live cell image were generated. A schematic illustration of both U-net approaches is visualized in Fig. 12.

#### Canny method

For conventional edge detection, we applied the Canny method [38]. Customized image processing software based on the Canny method was implemented by using MATLAB’s image processing toolbox [*edge* (*Image*,*‘canny’*]). First, images were converted to grayscale (*rgb2gray*). The relevant edges between cell and background were isolated by manually adjusting a threshold value. These edges were dilated by a user defined factor typically in the range of 1 to 20 pixel. Finally, smaller elements were removed and the resulting areas were eroded again, resulting in a binary image consisting of the cell monolayer and the gap (using *bwareaopen* and *imerode*). The *bwboundaries* function from MATLAB was used to calculate the position and length of the edge.

#### Manual detection and segmentation

For training purposes and as a reference to the presented wound healing analysis methods, data were manually generated. In addition to the 280 manually segmented cell images for iCD training we manually segmented 1467 individual cells to evaluate the training success of the neural network. For comparison with automatic cell density determinations approximately 360 individual endothelial cells were manually marked. To validate the cell velocity estimation, the velocity of each cell was measured manually on two consecutive images. Again, this process was performed for 360 single cells. Furthermore, the manual cell tracking was repeated 3 times by different operators. Manual tracking was done by using a custom made MATLAB application. In this process, the user marked the cell in its centroid in two successive images.

For training and evaluation of the population scale methods the leading edge was detected three times manually. Of these, 170 images were used for training purpose and 50 for evaluation.

## Data Availability

The datasets used and/or analyzed during the current study are available from the corresponding author on reasonable request. The pre-trained MATLAB application will be uploaded on MATLAB file exchange or can be requested from the corresponding author via email.

## Abbreviations

iCD: Intelligent Cell Detection
CIV: Cell Image Velocimetry
CNN: Convolutional Neural Networks
iDSD: Intelligent direct scratch detection
HeLa: Cell line originated from Henrietta Lacks
GUI: Graphical User Interface
